# How are compassion fatigue, burnout, and compassion satisfaction affected by quality of working life? Findings from a survey of mental health staff in Italy

**DOI:** 10.1186/s12913-017-2726-x

**Published:** 2017-11-21

**Authors:** Gaia Cetrano, Federico Tedeschi, Laura Rabbi, Giorgio Gosetti, Antonio Lora, Dario Lamonaca, Jill Manthorpe, Francesco Amaddeo

**Affiliations:** 1 Social Care Workforce Research Unit, King’s Policy Institute, King’s College London, London, UK; 2 Department of Neurosciences, Biomedicine and Movement Sciences, Section of Psychiatry, University of Verona, Verona, Italy; 3 Department of Human Sciences, University of Verona, Verona, Italy; 4 Mental Health Department, Azienda Sociosanitaria Territoriale Lecco, Lecco, Italy; 5 Mental Health Department, Azienda ULSS 9 Scaligera, Legnago, Italy

**Keywords:** Compassion fatigue, Burnout, Compassion satisfaction, Quality of working life, Mental health staff, Mental health services

## Abstract

**Background:**

Quality of working life includes elements such as autonomy, trust, ergonomics, participation, job complexity, and work-life balance. The overarching aim of this study was to investigate if and how quality of working life affects Compassion Fatigue, Burnout, and Compassion Satisfaction among mental health practitioners.

**Methods:**

Staff working in three Italian Mental Health Departments completed the Professional Quality of Life Scale, measuring Compassion Fatigue, Burnout, and Compassion Satisfaction, and the Quality of Working Life Questionnaire. The latter was used to collect socio-demographics, occupational characteristics and 13 indicators of quality of working life. Multiple regressions controlling for other variables were undertaken to predict Compassion Fatigue, Burnout, and Compassion Satisfaction.

**Results:**

Four hundred questionnaires were completed. In bivariate analyses, experiencing more ergonomic problems, perceiving risks for the future, a higher impact of work on life, and lower levels of trust and of perceived quality of meetings were associated with poorer outcomes. Multivariate analysis showed that (a) ergonomic problems and impact of work on life predicted higher levels of both Compassion Fatigue and Burnout; (b) impact of life on work was associated with Compassion Fatigue and lower levels of trust and perceiving more risks for the future with Burnout only; (c) perceived quality of meetings, need of training, and perceiving no risks for the future predicted higher levels of Compassion Satisfaction.

**Conclusions:**

In order to provide adequate mental health services, service providers need to give their employees adequate ergonomic conditions, giving special attention to time pressures. Building trustful relationships with management and within the teams is also crucial. Training and meetings are other important targets for potential improvement. Additionally, insecurity about the future should be addressed as it can affect both Burnout and Compassion Satisfaction. Finally, strategies to reduce possible work-life conflicts need to be considered.

## Background

While mental health professionals experience similar organizational stressors to other human service workers, the intrinsic characteristics of mental health work constitute further stress factors. Mental health professionals face additional emotional strain by the very nature of their work with distressed individuals, often over extended periods.

Some critical risk factors seem to be particularly applicable to mental health care and, among these, the most important is the relationship that mental health professionals have with clients who are experiencing psychological trauma [1]. Through exposure to clients’ reenactments and accounts of traumatic experiences, helping professionals are themselves vulnerable to a variety of effects and symptoms, including depression, anxiety, intrusive imagery, numbing and avoidance phenomena, cognitive shifts, as well as social and relational problems [2–6]. Such negative consequences have been described in terms of Compassion Fatigue, which is defined as the practitioner’s reduced capacity to be empathic or bear the suffering of clients [2, 3, 7]. On the other hand, the positive outcome from working with challenging clients has been defined as Compassion Satisfaction. Compassion Satisfaction, which reflects the rewards of caring for others, has been identified as a possible element that counterbalances the risks of Compassion Fatigue [8]. However, Compassion Fatigue and Compassion Satisfaction seem to be negatively correlated, i.e. higher levels of Compassion Fatigue may overwhelm the professional’s sense of efficacy and prevent him/her from experiencing Compassion Satisfaction [8, 9].

Burnout is defined as “a state of physical, emotional, and mental exhaustion caused by long-term involvement in emotionally demanding situations” [10]. Unlike Compassion Fatigue, Burnout is not directly related to exposure to traumatic material [11]. Compassion Fatigue differs from Burnout in that the latter evolves gradually and is a result of emotional exhaustion. In contrast, Compassion Fatigue can emerge suddenly. Thus, Burnout does not appear to capture the effects of trauma as an occupational stressor. A study of social workers involved with clients who had some exposure to the events of September 11, 2001, confirmed that exposure to clients traumatized by the World Trade Center disaster increased Compassion Fatigue, but not Burnout. Furthermore, both Compassion Fatigue and Burnout appeared to be associated with psychological problems [11].

These aspects were selected as key variables in our study as they may be a potential threat to staff wellbeing and, consequently, care quality. Among the detrimental effects of Compassion Fatigue, Burnout and lack of Compassion Satisfaction there is evidence of high turnover rates, negative attitudes towards patients, lack of communication, and clinical errors [3, 11, 12].

Possible risk and protective factors for Compassion Fatigue, Burnout, and Compassion Satisfaction have been identified, including: socio-demographic characteristics, such as age, marital status, and education [13, 14]; coping styles [2, 3, 15]; exposure to trauma [13, 16, 17]; personal history of trauma [18, 19]; job-related factors, including workload [17], length of experience [19], and attendance at training [13]; and, finally, work environment factors [3, 17].

Although the literature on work-related stress (especially on Burnout) in the healthcare sector is substantial, to date there is a limited amount of literature examining the three dimensions of Compassion Fatigue, Burnout and Compassion Satisfaction together and including the wide range of mental health professional categories; instead most studies focus on only one or two professional categories at a time.

This paper reports the findings of a study that adopted an original approach in framing the three dimensions of Compassion Fatigue, Burnout, and Compassion Satisfaction within a comprehensive model of quality of working life. We defined quality of working life as an individual’s broad job-related experience. We applied a multidimensional approach to the study of quality of working life, similar to that developed by the European Foundation for the Improvement of Living and Working Conditions (EUROFOUND) [20–22]. Within this framework, two constitutive areas are recognized as characterizing quality of working life: the intrinsic quality of work and the quality of work-life balance [23–26]. The intrinsic quality of work derives from the relationships between the worker’s needs and the intrinsic characteristics of the work. In this present study, six dimensions (economic, ergonomic, complexity, autonomy, control, and symbolic dimension) for the analysis of the intrinsic quality of work were included, which in turn were further subdivided into 11 indicators. Quality of work-life balance is about reconciling working life and life outside work. This is an essential condition both for encouraging entry into the labour market and for enabling people to remain at work [20]. Work-life balance was analysed in this study through two indicators (i.e., impact of work on life and impact of life on work), in order to capture bi-directional interconnections between work and life.

Against this background, the overarching aim of this study was to investigate whether and to what extent the quality of working life as perceived by mental health staff affects levels of Compassion Fatigue, Burnout, and Compassion Satisfaction.

## Methods

### Study sites and population

Three centres in Northern Italy were selected for this cross-sectional study based on their willingness to participate: Lecco Mental Health Department (MHD) (Lombardy Region), Legnago MHD and South Verona Community Mental Health Service (CMHS) (Veneto Region). Lecco MHD serves a population of 336,127 inhabitants and is responsible for planning and coordinating all mental health services in the Province of Lecco. Legnago MHD serves a population of 154,015 inhabitants. South Verona CMHS serves a catchment area of 108,421 inhabitants and is one of the four CMHSs constituting the Verona MHD. All three sites contain community mental health centres, day-hospital/day care rehabilitation centres, general hospital inpatient psychiatric units, and non-hospital residential care with medium- and long-term facilities, as required by Italian national mental health policy [27]. Since mental health services in the three sites share similar organizational features, the analysis considered the three centres together. There was no selection of participants and all members of staff undertaking clinical and care activities were invited to complete a survey, including psychiatrists, psychiatrists in training, psychologists, social workers, psychiatric nurses, educators, rehabilitation therapists, and healthcare support workers. Data were collected between March and September 2014.

### Measures

#### Independent variables

Socio-demographics, occupational characteristics, and 13 measures of quality of working life, collected through the Quality of Working Life Questionnaire [28], were included as independent variables.

Data on socio-demographic characteristics covered: age, gender, marital status, living situation, and educational level.

The variables measuring occupational characteristics were: centre, profession/job role, seniority (i.e., total number of years worked in the current mental health service and, when applicable, in other health or social services), employing organization (i.e., local health authority/hospital trust, university, not for profit organization), type of contract (open-ended vs. fixed-term contract), and time dedicated to clinical and care work (i.e., percentage of working hours dedicated to clinical and care work).

Moreover, the analysis included 10 composite indicators and 3 single-item indicators to assess the quality of working life as perceived by mental health staff. The conceptual structure of these indicators as detailed elsewhere [25] has been developed on the basis of the framework used in the European Working Conditions Surveys (EWCS) designed by EUROFOUND [20]. Table 1 sets out indicators of quality of working life with specific items defined.

**Table 1:** Indicators of quality of working life

Indicator	Indicator
Opinion of current salary	Participation
- Insufficient/Sufficient/Good	In the previous 12 months, participated in meetings to: - Discuss the service organization - Define methods, technologies, and techniques - Plan and verify the activities of the service - Decide projects and services to be realized
Attended training events in the past 24 months	Perceived quality of meetings
- Yes vs. No	Meetings give me the opportunity to: - Discuss interesting issues - Bring my contribution - Be appreciated for my contribution - Influence the decisions of the group - Adequately deal with problems - Receive emotional support
Perceived need of training	Organizational commitment
- Yes vs. No	The group: - Has a clear idea of the mission of the service - Feels the mission of the service as its own - Is involved in the achievement of the objectives - Critically reflects on its work - Receives support from managers in the development of its own ideas and projects
Ergonomic problems	Impact of work on life
- Hot or cold temperatures - Excessive or insufficient light levels - Inappropriate workspace - Poor hygiene - Shortage of personal protective equipment - Lifting, pushing or pulling heavy loads - Time pressures (work pace and intensity) - Long periods of time at computer - Risk of assaults	How work influences: - One’s caregiving role (taking care of children, parents, etc.) - One’s home duties (housekeeping, etc.) - One’s hobbies and interests (friends, sport, etc.) - One’s political, social and religious activities
Job complexity	Impact of life on work
- Different tasks to be carried out - Unexpected situations and problems to solve - Tasks requiring experience - Tasks with high levels of responsibility - Job rotation with colleagues - Coordination of other people’ s work	How life influences: - One’s career opportunities - One’s professional growth - One’s opportunity to accept extra duties - One’s opportunity to change job
Trust	Perceived risks for the future
- The manager knows well my tasks - I am free in the work and little controlled - The manager helps me when I have problems - Colleagues help me when I have problems - The manager trusts me	Perceived risk for the future of: - Losing job - Having an inadequate work competence - Being unable to maintain one’s family - Being unable to afford a substantial item - Not receiving an adequate pension
Autonomy	
- I can decide to have breaks during work - I can decide how to do the work - I have autonomy in solving an organizational problem - I can decide the timing of my work - I can decide the paucity and intensity of my work - I can choose the tasks to carry out - I can make weekly or monthly plans - I can decide the quality level of my work	

Such indicators have been used on large samples in other Italian work settings including craftwork and agriculture [28, 29], and were pre-tested with a sample of 16 mental health staff in South Verona covering different professional groups (i.e., psychiatrists, psychologists, social workers, nurses, psychiatrists in training, educators, and healthcare support workers).

As for perceived risks for the future, participants were asked to select up to 5 pre-determined types of risks; responses were then coded into three groups based on the number of items selected (i.e., 0 items = no risks; 1 or 2 items = some risks; 3, 4 or 5 items = many risks).

As for the other composite indicators, the average scores for each indicator were considered, with higher scores indicating greater values of that measure. Items related to ergonomic problems (including time pressures), job complexity, trust, autonomy, perceived quality of meetings, were rated on five-point Likert scales from 1 (never) to 5 (always). Participation was rated on a three-point Likert scale from 1 (never) to 3 (often). Impact of work on life, impact of life on work and organizational commitment were rated on a five-point Likert scale from 1 (none) to 5 (very much).

Furthermore, the survey asked about the following three single-item indicators: opinion of current salary (insufficient, sufficient, and good), attendance at training events in the past 24 months, and whether he/she perceived a need to attend future training events.

Discriminant and convergent validity were evaluated. As far as discriminant validity is concerned, Pearson correlation coefficients among quality of working life indicators were calculated. The results showed only weak or moderate associations, with coefficients below 0.50 in absolute value in all cases apart from those between trust and quality of meetings (0.51) and of these measures with organizational commitment (0.52 in both cases), highlighting our indicators referred to distinct constructs.

As for convergent validity, Cronbach’s α coefficient of internal consistency reliability was calculated for all the above indicators, except for perceived risks for the future since the items were not meant to measure a single unidimensional construct. The coefficients ranged from 0.62 (job complexity) to 0.87 (perceived quality of meetings), highlighting that the composite indicators showed either acceptable (in a few occurrences) or good (in most cases) internal consistency.

Finally, the risk of redundancy of our indicators as predictors in a regression was evaluated via computation of the Variance Inflation Factor, a measure of the increase in variance of the estimated regression coefficients due to the presence of the other regressors [30]. The latter ranged between 1.06 and 1.75, thus showing only weak or moderate multicollinearity. Descriptive statistics of the above indicators have been published elsewhere [31].

#### Dependent variables

Compassion Fatigue (CF), Burnout (BO), and Compassion Satisfaction (CS) were chosen as outcomes of the above variables. The Professional Quality of Life Scale (ProQOL III) was used to measure CF, BO, and CS. The Italian version of this instrument has been validated [32]. Alpha scores range from .72 (BO) to .80 (CF) and .87 (CS), indicating adequate internal consistency. It has been demonstrated that the scale has good construct validity, and there is evidence that this version of the measure reduced the known collinearity between CF and BO [32]. A recent study by Galiana and colleagues [33] offered further evidence of the existence of the three factors identified by Stamm [34].

The ProQOL III comprises 30 items corresponding to the three subscales: CF, BO, and CS scales. Participants were asked to indicate how often (from 0 = never to 5 = very often), during the last 30 days, each item was experienced. The 0 responses were then recoded into 1, as the 1–5 Likert scale was considered more familiar and made it possible to overcome the issues related to reversing the 0 score. Then, the value for each subscale was calculated as the sum of the values of each item.

All instruments were administered using a Paper and Pencil Interview (PAPI) system.

### Statistical analysis

Descriptive and inferential analyses were performed in order to describe the participants’ characteristics and the associations between indicators of quality of working life and levels of CF, BO, and CS and finally to predict such subscales. In order to minimize the risk of bias, a decision was made to discard cases with more than 25% missing items in the Quality of Working Life Questionnaire (weights inversely proportional to the number of items of the indicator were used to calculate the percentage of missing values), or with more than 15 (out of 30) missing values in the ProQOL III. In the resulting dataset, the Corrected Item Mean Substitution method [35] was used (i.e. the item mean across participants weighted by the subject’s mean of completed items) to impute missing ProQOL III values, and in all parametric analyses, for each subscale, weights proportional to the number of completed items were used. Absolute frequencies and percentages were computed for socio-demographics and occupational variables. The mean values, standard deviations (SD), ranges and quartiles of CF, BO, and CS scores were also calculated.

A separate bivariate analysis was performed in order to identify which of the indicators of quality of working life were associated with CF, BO, and CS. The Spearman’s rank correlation was calculated for continuous indicators, and its significance was assessed by t-test. Moreover, the mean values and related standard errors (SEM) of CF, BO, and CS, together with absolute frequencies and percentages were computed for each occurrence of categorical variables, and the statistical significance of the dependence between them and the outcome variables was assessed by the Kruskal-Wallis test.

A hierarchical (weighted) multiple regression was performed for CF, BO, and CS using sociodemographic and occupational variables as the first block of predictors and quality of working life indicators as the second. The selection of variables for the first block was performed using the “leaps and bound” algorithm [36] in order to identify the subset of variables with the highest adjusted R^2^. As for the second block, the choice of the variables was based on statistical significance in bivariate regressions (overall significance was considered for categorical variables). The adjusted R^2^ of the final regressions was also computed, in order to highlight the increase in the predictive power of our models once working life variables were included. Given the bounded nature of the outcomes, a robustness check for the sensitivity of results to the model assumptions was performed. In particular, fractional logit models (i.e., regressions with a logit link on the standardized values of the outcomes) [37] using the same weights and the same variables of the final linear regressions were performed. Finally, the correlation between the outcome and its predicted value using single items of significant quality of working life indicators was calculated for each regression, in order to assess which single aspects were most strongly associated with CF, BO, and CS. Statistical analyses were performed using Stata Release 14.1 [38].

## Results

The total number of staff invited to complete the survey was 461, of whom 416 responded to both questionnaires. Among them, 16 returns were excluded due to the exclusion criteria related to the amount of missing values, thus 400 surveys were retained for analysis, representing a response rate of 87%. Table 2 shows participants’ socio-demographic and occupational characteristics.

**Table 2:** Participants’ socio-demographic and occupational characteristics (*n* = 400)

	Number	Percent
Gender		
Male	95	24.1
Female	299	75.9
Age		
18–29 years	35	8.9
30–39 years	105	26.8
40–49 years	134	34.2
50–59 years	102	26.0
> 60 years	16	4.1
Marital status		
Single	111	28.5
Married	239	61.3
Separated, Divorced, Widowed	40	10.3
Living Situation		
Alone	75	19.1
With partner or family or other	317	80.9
Educational level		
None/Primary/Lower secondary school	17	4.3
Professional qualification/High school diploma	154	39.0
University diploma or degree	134	33.9
Master/PhD/Specialization	90	22.8
Centre		
South Verona	132	33.0
Legnago	66	16.5
Lecco	202	55.5
Profession/job role		
Psychiatrist/Psychiatrist in training	79	19.9
Psychologist	27	6.8
Nurse	121	30.5
Educator/Social worker	65	16.4
Rehabilitation therapist	22	5.5
Healthcare support worker	83	20.9
Employing Organization		
Local health authority/Hospital trust	249	62.4
University	40	10.0
Not for profit organization	110	27.6
Type of contract		
Open-ended contract	331	82.75
Fixed-term contract	69	17.25
Years of experience in the health and care sector		
1–10 years	168	42.0
11–30 years	196	49.0
More than 30 years	36	9.0
% of time dedicated to clinical and care work		
Up to 50%	59	15.7
From 50% to 80%	152	40.5
More than 80%	164	43.7

Overall, CF showed a mean of 14.4 (SD = 4.1), with scores of completed questionnaires ranging between 10 and 36; BO showed a mean of 22.9 (SD = 4.5) and a range of 10–39, while the mean value of CS was 32.7 (SD = 7.1), with scores ranging between 10 and 50. CF showed a first quartile of 11, a median score of 13 and a third quartile of 16; the quartiles for BO were 20, 23 and 25; finally, the CS scores had a lower quartile of 28, a median of 33 and an upper quartile of 38.

Tables 3 and 4 present the bivariate associations of the continuous and the categorical indicators of quality of working life respectively with the three outcome measures. Concerning CF, ergonomic problems and the two variables on work-life balance showed the strongest correlations (with higher values of such variables corresponding to increasing levels of CF, *p*-value < 0.001 in all cases). As for BO, the strongest associations were found with perceived quality of meetings and trust (in negative) and with ergonomic problems (in positive); other important associations were found with lower levels of organizational commitment and of autonomy, a higher impact of work on life (*p*-value < 0.001 in all the above-mentioned cases), and with perceiving more risks for the future (*p*-value = 0.001) and having a less positive opinion of current salary (*p*-value = 0.010). Finally, looking at CS, the most relevant correlations were found with higher levels of perceived quality of meetings and of organizational commitment, with important positive associations also observed with the indicators of autonomy and trust (*p*-value < 0.001 in all cases).

**Table 3:** Associations between (continuous) indicators of quality of working life and compassion fatigue, burnout, and compassion satisfaction^a^

	Compassion fatigue (*N* = 400)		Burnout (*N* = 400)		Compassion satisfaction (*N* = 400)	
	Coefficient	*p*-value	Coefficient	*p*-value	Coefficient	*p*-value
Ergonomic problems	**0.23**	**<0.001**	**0.27**	**<0.001**	**−0.12**	**0.012**
Job complexity	**0.13**	**0.009**	0.02	0.650	0.08	0.107
Trust	−0.08	0.126	**−0.30**	**<0.001**	**0.20**	**<0.001**
Autonomy	−0.04	0.449	**−0.20**	**<0.001**	**0.20**	**<0.001**
Participation	0.07	0.156	**−0.10**	**0.037**	**0.15**	**0.003**
Perceived quality of meetings	**−0.13**	**0.013**	**−0.31**	**<0.001**	**0.29**	**<0.001**
Organizational commitment	−0.02	0.749	**−0.24**	**<0.001**	**0.28**	**<0.001**
Impact of work on life	**0.24**	**<0.001**	**0.19**	**<0.001**	−0.10	0.052
Impact of life on work	**0.18**	**<0.001**	0.09	0.067	−0.02	0.711

^a^Spearman’s rank correlations; significance assessed by t-test. Bold indicates effect size with statistical significance

**Table 4:** Associations between (categorical) indicators of quality of working life and compassion fatigue, burnout, and compassion satisfaction^a^

		Compassion fatigue		Burnout		Compassion satisfaction	
	N (%)	Mean (SEM)	Chi-square (*p*-value)	Mean (SEM)	Chi-square (*p*-value)	Mean (SEM)	Chi-square (*p*-value)
Attended training events in the past 24 months							
Yes	387 (97.2)	14.4 (0.2)	0.1 (0.753)	22.8 (0.2)	<0.001 (0.987)	32.7 (0.4)	0.8 (0.370)
No	11 (2.8)	14.7 (1.4)		23.3 (2.0)		29.4 (2.4)	
Perceived need of training							
Yes	372 (93.2)	14.4 (0.2)	0.005 (0.942)	22.9 (0.2)	0.02 (0.878)	32.9 (0.4)	**5.1 (0.024)**
No	27 (6.8)	14.3 (0.8)		23.0 (1.1)		28.9 (1.7)	
Perceived risks for the future							
No risks	36 (9.0)	12.7 (0.4)	**6.9 (0.032)**	20.5 (0.5)	**13.9 (0.001)**	36.0 (0.8)	**10.0 (0.007)**
Some risks	182 (45.7)	14.4 (0.3)		22.7 (0.3)		32.3 (0.5)	
Many risks	180 (45.2)	14.7 (0.3)		23.5 (0.4)		32.4 (0.6)	
Opinion of current salary							
Insufficient	89 (22.1)	13.9 (0.4)	3.1 (0.209)	23.8 (0.5)	**9.2 (0.010)**	31.3 (0.8)	5.9 (0.052)
Sufficient	216 (54.1)	14.7 (0.3)		23.1 (0.3)		33.0 (0.5)	
Good	95 (23.8)	14.2 (0.4)		21.6 (0.4)		33.3 (0.6)	

^a^Significance assessed by Kruskal-Wallis test. Bold indicates effect size with statistical significance

The results of the multivariate analysis are shown in Table 5. Variables that turned out significant in simple linear regressions were: for CF, ergonomic problems, job complexity, trust, perceived quality of meetings, impact of work on life and of life on work, and perceived risks for the future; in the case of BO, ergonomic problems, trust, autonomy, participation, perceived quality of meetings, organizational commitment, impact of work on life, perceived risks for the future and opinion on current salary; finally, for CS, ergonomic problems, trust, autonomy, participation, perceived quality of meetings, organizational commitment, impact of work on life, perceived need of training and risks for the future. After controlling for the other covariates, the regression models with CF and BO as outcomes showed that ergonomic problems were highly related to both (*p*-value equal to 0.008 in the former case and 0.002 in the latter one), with impact of work on life also emerging as a predictor of negative outcomes for CF (*p*-value = 0.009) and BO (*p*-value = 0.007). Finally, impact of life on work turned out to be a further predictor of CF (*p*-value = 0.011), while perceiving more risks for the future (*p*-value = 0.001) and lower levels of trust (*p*-value = 0.004) were associated with BO in multivariate analysis. As for CS, a highly significant association with perceived quality of meetings was found (*p*-value = 0.007), with need to attend training (*p*-value = 0.035) and perceiving no risks for the future (*p*-value = 0.012) also predicting higher CS levels. Fractional logit models (results not shown) led to similar *p*-values with respect to linear regressions, with direction of associations (except for participation in the CS regression, but with parameters close to 0 and non-significant in both cases) and significant results confirmed, further strengthening our findings. Moreover, organizational commitment switched from marginally non-significant to marginally significant (*p*-value = 0.049) in the regression related to CS, and perceived risks for the future turned out as significant in the regression on CF (*p*-value = 0.021). The comparison of the best subset regressions using socio-demographics and occupational variables with the final regressions highlighted that most of the predictive power of our models was accounted for by quality of working life indicators. In fact, adjusted R^2^ increased from 0.01 to 0.15 in the case of CF, from 0.06 to 0.26 for BO, and from 0.06 to 0.18 in the regression having CS as outcome, showing that socio-demographics and occupational variables played a minor role, while variables related to job-related experience strongly increased the predictive power of our regression models.

**Table 5:** Indicators of quality of working life predicting compassion fatigue, burnout, and compassion satisfaction^a^

	Compassion fatigue (*N* = 362)		Burnout (*N* = 360)		Compassion satisfaction (*N* = 360)	
	Adjusted R^2^ = 0.15		Adjusted R^2^ = 0.25		Adjusted R^2^ = 0.18	
	Coeff.	95% CI	Coeff.	95% CI	Coeff.	95% CI
Ergonomic problems	**1.063**	(0.278, 1.848)	**1.228**	(0.438, 2.017)	0.160	(−1.142, 1.463)
Job complexity	0.501	(−0.298, 1.300)	–	–	–	–
Trust	−0.461	(−1.202, 0.279)	**−1.210**	(−2.038, −0.382)	0.454	(−0.907, 1.816)
Autonomy	–	–	−0.241	(−0.955, 0.474)	0.173	(−1.011, 1.356)
Participation	–	–	−0.021	(−0.869, 0.828)	−0.010	(−1.400, 1.380)
Perceived quality of meetings	−0.618	(−1.360, 0.123)	−0.726	(−1.550, 0.098)	**1.873**	(0.529, 3.217)
Organizational commitment	–	–	−0.295	(−1.113, 0.523)	1.328	(−0.030, 2.687)
Impact of work on life	**0.683**	(0.175, 1.191)	**0.692**	(0.194, 1.190)	−0.685	(−1.507, 0.137)
Impact of life on work	**0.622**	(0.141, 1.103)	–	–	–	–
Perceived need of training (Yes vs. No)	–	–	–	–	**3.138**	(0.171, 6.106)
Perceived risks for the future						
Some risks vs. No risks	0.818	(−0.629, 2.265)	**1.589**	(−0.010, 3.189)	**−3.003**	(−5.559, −0.446)
Many risks vs. No risks	1.261	(−0.233, 2.755)	**2.826**	(1.112, 4.541)	**−3.170**	(−5.880, −0.460)
Opinion of current salary						
Sufficient vs. Insufficient	–	–	−0.035	(−1.129, 1.058)	–	–
Good vs. Insufficient	–	–	−0.419	(−1.836, 0.997)	–	–

^a^Multiple regressions controlling for socio-demographic and occupational variables. Bold indicates effect size with statistical significance

Finally, when looking at the single items included in the indicators of quality of working life that turned out to be significant in the regressions, the strongest association was found with the item of time pressures as part of the ergonomic problems variable. More specifically, an increase in the BO score was found for people who perceived higher levels of time pressures, with a correlation coefficient of 0.32.

## Discussion

This study analysed in depth a core set of indicators of quality of working life and their role in affecting levels of Compassion Fatigue, Burnout, and Compassion Satisfaction among mental health staff.

Beginning with Compassion Fatigue and Burnout, one of the strongest associations was with ergonomic problems. In our models the experience of ergonomic problems significantly increased the levels of both outcomes. The presence of additional strains in the environment seems to have a negative impact on the ability to exert one’s role as a helper, thus exacerbating levels of both Compassion Fatigue and Burnout. This is consistent with previous research; Abu-Bader [14] found that higher satisfaction, lower burnout and lower turnover were a function of adequate working conditions. Interestingly, among the ergonomic problems investigated in this study, those that seemed to have a stronger impact, especially on Burnout, were time pressures. Time pressures are likely to generate a feeling of being overloaded as well as reducing the possibility of debriefing and taking time off, thus creating a vicious circle where the risk of Burnout can seriously increase [7].

Additionally, increased levels of Compassion Fatigue and Burnout were predicted by higher levels of impact of work on life. This finding indicates that work-life balance is essential in preventing work-related negative outcomes, as various authors assert [7, 39]. The importance of reconciliation of work and private life for the quality of work and employment was acknowledged in the Europe 2020 strategy [40]. Our finding that the impact of work on life was related to stronger negative consequences than impact of life on work (which was significant only in relation to Compassion Fatigue) seems to show that participants were more concerned about work interference with their personal life than the reverse (for a review of studies on the interactions between personal life - especially family relationships - and quality of work life, see Colichi and colleagues [41]).

As for Burnout only, our results showed that trust predicted lower levels of such outcome. This result is in line with research demonstrating that receipt of support and feelings of being trusted by managers and co-workers are critical in protecting against Burnout [42–44].

When looking at factors influencing Compassion Satisfaction, we found three significant explanatory factors, i.e. perceived need of training, perceived quality of meetings, and perceived risks for the future. These factors share similar features in that they reflect aspects of workers’ motivation and engagement, and differentiate from the others analysed as they seem potentially able to make a difference in a positive way. Personal and predisposing factors may be involved. Indeed, staff who are more motivated and engaged per se are more likely to perceive meetings as more useful and to invest in developing their skills and knowledge, consequently deriving more enjoyment from taking part in meetings and being keener to receive training. Moreover, a higher motivation could lead to be less concerned about future risks (e.g., losing job, having inadequate work competence, being unable to maintain one’s family, being unable to afford a substantial item, receiving an inadequate pension). However, due to the cross-sectional nature of our study, it is also possible to see motivation as an effect rather than a cause: in particular, since team meetings and training serve several important functions (as they potentially enable professionals to improve their skills, exert control over their work routines and give each other support), their quality could increase staff motivation, while feeling insecure about one’s future might affect staff motivation and investment in work. Therefore, although we may not draw any conclusion about the quality of either meetings or training perceived by our participants, both meetings and training should be considered by managers and team leaders as a way to improve Compassion Satisfaction.

Interestingly, perceived risks and uncertainties were associated with Burnout too, further suggesting the negative effects of job insecurity on staff wellbeing [20–22, 45]. The experience of insecurity for the future may be regarded as a ‘catalyst’ element, i.e. it is significant in affecting both positive (Compassion Satisfaction) and negative (Burnout) outcomes. Thus, additional attention should be paid to this factor as its spectrum of action seems broader than that of other variables. Indeed, all other significant variables in the final regressions were statistically associated either with Compassion Fatigue and/or Burnout or with Compassion Satisfaction, but not with both. For example, ergonomic problems seemed to play an important role for negative but not necessarily for positive outcomes (despite being associated with Compassion Satisfaction in bivariate analysis, no significant association with such outcome was found in the regression). It may be argued that an adequate working environment is necessary in order to prevent negative consequences such as Compassion Fatigue and Burnout, but it is not a crucial factor in increasing levels of Compassion Satisfaction.

The strengths and limitations of this study should be acknowledged. The main strength of the study is the relatively large number of participants and the inclusion of multiple centres and of different mental health professionals working in varied settings, such as inpatient psychiatric units, outpatient, day, and residential services. In addition, the response rate was high. A further strength of the study is the inclusion of a large set of variables measuring socio-demographics, occupational characteristics and a core set of indicators of quality of working life. The latter made it possible to capture the subjective experience and perceptions of participants about their quality of working life as a whole. However, some limitations of the study should be taken into account. First, the cross-sectional nature of the survey did not allow the determination of causality. Moreover, as only centres from two regions in Northern Italy were included, the study results do not necessarily generalize to the whole country. Furthermore, because this study emphasized the role of organizational and environmental factors, other possibly relevant variables were not included in the survey. For example, individual coping strategies with work-related stresses were not considered, although extensive literature demonstrates their crucial role in tackling difficult situations at work [46–48]. Finally, this study analysed outcomes related to staff’s perception in mental health settings; future studies could integrate such results by considering different organisational settings and/or by including clients’ measures of effectiveness of care as main outcomes.

## Conclusions

These findings are potentially useful for health managers and team leaders in identifying factors affecting the ability to be compassionate and the risk of Burnout. It is widely recognized that lack of Satisfaction, Compassion Fatigue and Burnout have adverse consequences on work performance and service quality. Compassion Fatigue is likely to result in problems such as misjudgements, clinical errors and poor treatment planning, all serious issues for effective care [3, 11, 12]. Therefore, in order to provide adequate mental health services, managers need to provide their employees with adequate ergonomic conditions, paying special attention to time pressures. Building trustful relationships with management and within teams is also crucial. Training and meetings are other important targets for potential improvement, although how meetings and the need for training are perceived might depend on the motivation of members of staff themselves. Additionally, the issue of insecurity for the future should be taken into serious consideration as it can affect both Burnout and Compassion Satisfaction. Lastly, to positively influence their clients’ wellbeing, managers, administrators, and staff themselves need to consider strategies to reduce possible work-life conflicts.

## Abbreviations

95% CI: 95% Confidence interval
BO: Burnout
CF: Compassion fatigue
CMHS: Community mental health service
CS: Compassion satisfaction
EUROFOUND: European foundation for the improvement of living and working conditions
EWCS: European working conditions surveys
MHD: Mental health department
SD: Standard deviation
SEM: Standard error of the mean
